# Lipoic Acid Metabolism of *Plasmodium* - A Suitable Drug Target

**DOI:** 10.2174/138161212801327266

**Published:** 2012-08

**Authors:** Janet Storm, Sylke Müller

**Affiliations:** Wellcome Trust Centre for Molecular Parasitology, Institute of Infection, Immunity & Inflammation, College of Medical, Veterinary and Life Sciences, University of Glasgow, 120 University Place, Glasgow G12 8TA, UK

**Keywords:** Lipoic acid salvage, lipoic acid biosynthesis, malaria, vaccine development, chemotherapy.

## Abstract

α-Lipoic acid (6,8-thioctic acid; LA) is a vital co-factor of α-ketoacid dehydrogenase complexes and the glycine cleavage system. In recent years it was shown that biosynthesis and salvage of LA in *Plasmodium *are necessary for the parasites to complete their complex life cycle. LA salvage requires two lipoic acid protein ligases (LplA1 and LplA2). LplA1 is confined to the mitochondrion while LplA2 is located in both the mitochondrion and the apicoplast. LplA1 exclusively uses salvaged LA and lipoylates α-ketoglutarate dehydrogenase, branched chain α-ketoacid dehydrogenase and the H-protein of the glycine cleavage system. LplA2 cannot compensate for the loss of LplA1 function during blood stage development suggesting a specific function for LplA2 that has yet to be elucidated. LA salvage is essential for the intra-erythrocytic and liver stage development of *Plasmodium *and thus offers great potential for future drug or vaccine development.

LA biosynthesis, comprising octanoyl-acyl carrier protein (ACP) : protein N-octanoyltransferase (LipB) and lipoate synthase (LipA), is exclusively found in the apicoplast of *Plasmodium *where it generates LA *de novo *from octanoyl-ACP, provided by the type II fatty acid biosynthesis (FAS II) pathway also present in the organelle. LA is the co-factor of the acetyltransferase subunit of the apicoplast located pyruvate dehydrogenase (PDH), which generates acetyl-CoA, feeding into FAS II. LA biosynthesis is not vital for intra-erythrocytic development of *Plasmodium*, but the deletion of several genes encoding components of FAS II or PDH was detrimental for liver stage development of the parasites indirectly suggesting that the same applies to LA biosynthesis.

These data provide strong evidence that LA salvage and biosynthesis are vital for different stages of *Plasmodium *development and offer potential for drug and vaccine design against malaria.

## 1 MALARIA

Malaria is one of the most devastating tropical diseases caused by intracellular, protozoan parasites of the genus *Plasmodium. *Five species of *Plasmodium* (*P. falciparum, P. vivax, P. ovale, P. malariae* and *P. knowlesi*) cause disease in humans and infection with *P. falciparum*, the most deadly of these parasites, is responsible for more than 1 million deaths annually, primarily affecting children under the age of 5 and pregnant women in sub-Saharan Africa and Southeast Asia [1-4]. This is mainly due to emerging resistance against existing chemotherapies and the lack of an effective vaccine [5-7]. Artemisinin combination therapy (ACT) is the WHO recommended treatment for malaria, but treatment failures have been recently reported in Cambodia, which is a worrying situation. 

Therefore, expanding the lifespan of currently available antimalarials is of utmost importance [8, 9]. In addition, it is imperative that new targets for the development of antimalarials are identified and validated in order to pave the way for the generation of new effective and affordable chemotherapies to combat the disease. 

The genomes of several *Plasmodium *species have been sequenced, revealing a number of peculiarities in the parasite’s metabolism that might be exploitable for the development of new antimalarials [10-12]. Some metabolic pathways are lost from the parasite’s genome, such as the *de novo* biosynthesis of amino acids, while other parasite-specific features that allow host cell invasion and remodelling of the host cell after invasion, to maximise and guarantee parasite survival, have been expanded. One important discovery was that *Plasmodium *and other apicomplexan parasites harbour a non-photosynthetic plastid, called apicoplast, that originates from secondary endosymbiosis of an algae [13-15]. Metabolic functions associated with the apicoplast have attracted considerable interest with respect to identify processes vital to the parasite and absent from the human host, which are potentially exploitable for disease intervention. The type II fatty acid biosynthesis (FAS II) and non-mevalonate isoprenoid biosynthesis pathways have been under intense investigation and the latter has been successfully shown to be suitable for antimalarial drug development [16-19].

The life cycle of *P.* *falciparum* in its two hosts, the *Anopheles *mosquito and the human, is complex (http://www.cdc.gov/malaria/-about/biology/index.html) and requires its metabolism to adapt to changes in temperature, pH and nutritional sources. These fluctuations clearly lead to alterations in the metabolic activities and requirements of the parasites, and therefore suitable and exploitable targets for drug development that are specific for the life cycle stages of the parasite are likely to be identified [9]. 

Humans are infected through the infective bite of a female *Anopheles* mosquito which transmits infective sporozoites; these invade hepatocytes where they multiply into thousands of merozoites that are released into the bloodstream. This part of the *P.* *falciparum *life cycle is asymptomatic but its interruption would have great impact on the pathology and spread of the disease. 

Merozoites released from the liver infect erythrocytes and asexually multiply within 48 or 72 hours (depending on the human *Plasmodium *species) into 16 to 32 daughter merozoites that are released into the blood stream to infect other erythrocytes. This periodic release of merozoites causes the typical fever bouts observed in malaria patients. The intra-erythrocytic part of the life cycle causes the clinical symptoms and pathology of the disease and can be lethal and is the one targeted by most of the existing chemotherapies to cure the disease. However, only few antimalarials efficiently reduce or prevent the formation of gametocytes, one of the pre-requisites to prevent transmission of the disease [20, 21].

The third part of parasite development is the sexual cycle which is initiated by the generation of male and female gametocytes in the human host, and which is triggered by cues that are not yet fully understood. Unlike asexual parasites, the gametocytes are infective to the *Anopheles* vector and therefore essential for the transmission of the parasites from human to vector. Once taken up by the mosquito, the gametocytes develop into male and female gametes and upon fertilisation the zygote matures into a motile form called the ookinete. The ookinete migrates through the midgut wall into the basal lamina of the mosquito’s midgut, where it forms the oocyst and where upon multiple sporogonic mitotic divisions, sporozoites develop. Infective sporozoites migrate into the salivary gland where they are primed to be injected into a new mammalian host. Transmission blocking agents are important to reduce transmission of the disease, but also to prevent spread of drug resistance and are therefore the focus of renewed interests and efforts to eradicate malaria [9].

This review will summarise the current knowledge about α-lipoic acid (LA) metabolism in *Plasmodium* and other related apicomplexa and discusses its potential as a drug target. The components of the LA metabolic pathway may be exploitable for the design of preventative, curative as well as transmission blocking agents. 

## 2 LIPOIC ACID

**Fig. (1):**
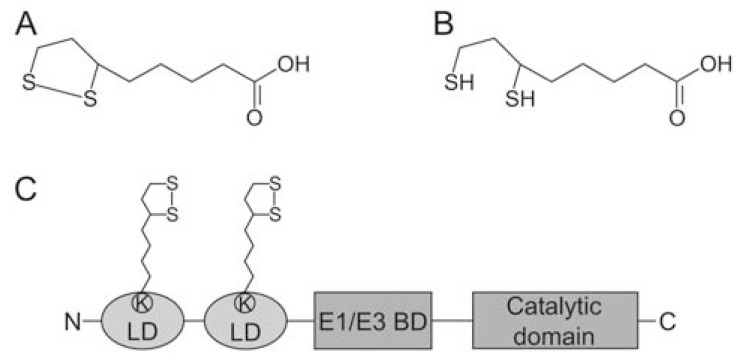
**Structure of lipoic acid and the E2-subunit of pyruvate dehydrogenase.** Chemical structure of free α-lipoic acid (LA) in its (**A**) oxidised and (**B**) its reduced form, dihydrolipoic acid (DHLA). (**C**) Schematic diagram of the E2-subunit of *P. falciparum* and human PDH depicting the two lipoyl-domains (LD), the E1/E3 binding domain (E1/E3 BD) and the catalytic domain of the protein. LA is covalently attached to a conserved lysine (K) residue of both LD.

LA is a cyclic disulphide containing derivative of octanoic acid that exists in oxidised and reduced form, a property integral to its functions (Fig. **1**). At a pH above 4.7, LA is de-protonated and negatively charged to form lipoate, the predominant form present under physiological conditions. It is an essential cofactor of the three α-ketoacid dehydrogenase complexes (KADH), namely pyruvate dehydrogenase (PDH), α-ketoglutarate dehydrogenase (KGDH) and branched chain α-ketoacid dehydrogenase (BCDH) as well as the glycine cleavage system (GCS), which are involved in energy and amino acid metabolism and are vital for cellular function [22, 23]. A fifth enzyme complex with a similar structure to PDH is also modified by lipoylation. Acetoin dehydrogenase is found in bacteria and is involved in the catabolism of acetoin [24]. 

Apart from its role in intermediate metabolism, LA has been shown to act as antioxidant; the free dihydrolipoic acid /lipoic acid (DHLA/LA) redox couple has a low redox potential of -0.32 V, which makes it a powerful reductant that reduces glutathione disulfide, vitamin C, vitamin E, as well as free radicals [25-29]. This property of the free redox couple also has therapeutic applications in diseases such as diabetes and Alzheimer’s disease [30-32]. Protein-bound lipoamide also plays a role as mitochondrial redox sensor and acts as antioxidant in the organelle [33-36]. Thus, LA has two major roles in the cell; it is involved in intermediate metabolism and it plays a pivotal role as redox sensor and antioxidant. 

## 3 α-KETOACID DEHYDROGENASE COMPLEXES AND THE GLYCINE CLEAVAGE SYSTEM

**Fig. (2):**
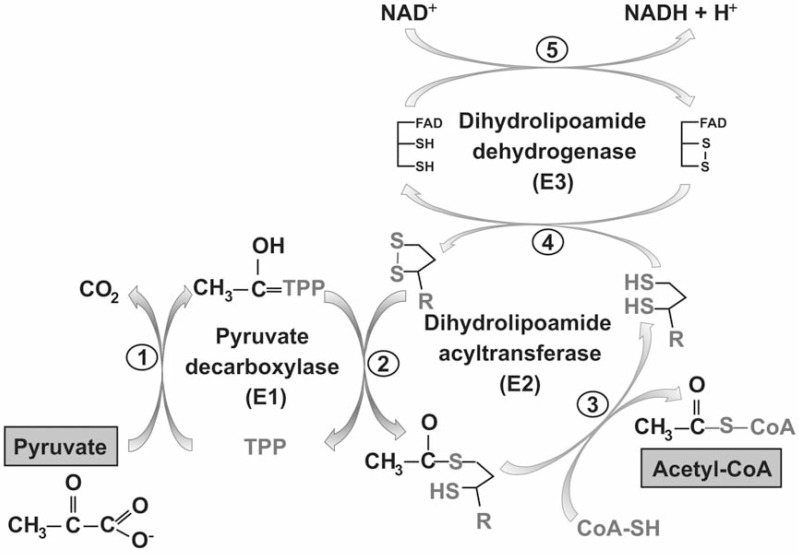
**Catalytic mechanism of PDH**. The reaction mechanism of the three catalytically active subunits of PDH (E1α/E1β forming a heterotetramer; E2 forming a 24- or 60-mer and E3, forming a dimer) is shown schematically. The E1-subunit decarboxylates pyuvate and the acetyl-moiety is covalently attached to the thiamine diphosphate co-factor (TPP) of E1. CO_2_ is released during the reaction (1). TPP transfers the acetyl-moiety to the oxidised lipoamide co-factor of E2 (2), which transfers it to CoA to form acetyl-CoA (3), which is released. E3 re-oxidises dihydrolipoamide (4) and NADH + H^+^ is generated (5).

The enzyme complexes that require LA as cofactor are the KADH and the GCS as outlined above. These multi-enzyme complexes are involved in amino acid and energy metabolism and consist of multiple copies of a substrate specific α-ketoacid decarboxylase (E1-subunit), an acyltransferase (E2-subunit) and a dihydrolipoamide dehydrogenase (E3-subunit). Generally, KADHs convert an α-ketoacid, NAD^+^ and coenzyme A (CoA) to CO_2_, NADH and acyl-CoA (Fig. **2**). The substrate specific E1-subunit contains thiamine pyrophosphate (TPP) as a cofactor and catalyses the decarboxylation of α-ketoacids, generating CO_2_ and acyl-TPP, the latter attached to the E1 protein. The acyl-group is then transferred to lipoamide, covalently bound to the E2-subunit via an amide linkage to a specific lysine residue. Lipoamide transfers the acyl-group from E2 to CoA, forming acyl-CoA, and is concurrently reduced. In the last step of the reaction lipoamide is re-oxidised by dihydrolipoamide dehydrogenase (E3), the FAD-dependent disulphide oxidoreductase leading to the formation of NADH (Fig. **2**) [23]. 

The E2-subunits of KADH form homo-trimers, which assemble to either 24-mers of octahedral organisation or 60-mers of icosahedral organisation [37]. These high molecular weight E2 multimers form the core of the multi-enzyme complexes to which the E1 and E3 subunits bind. The E2-subunit consists of three distinct domains; one to three lipoyl-domains, depending on the complex and the organisms it occurs in, the subunit binding domain and the catalytic domain (Fig. **1**). The lipoyl-domain contains the signature lysine residue that is post-translationally modified by lipoylation, the subunit binding domain confers binding of E1 and E3 and the catalytic domain transfers the acyl-moiety to CoA. [23, 37, 38]. The domains are separated by flexible linkers [39] which allows the lipoyl-domain to function as a swinging arm to transfer reaction intermediates between E1, E2 and E3 [23]. 

PDH catalyses the oxidative decarboxylation of pyruvate to generate acetyl-CoA. The enzyme complex is generally found in the mitochondrion where it links glycolysis with the tricarboxylic acid (TCA) cycle. Plants possess an additional PDH enzyme complex, which is present in the plastid, where it provides acetyl-CoA and NADH for fatty acid biosynthesis [40]. *Plasmodium* and other apicomplexan parasites possess a single PDH, which is exclusively present in the apicoplast [41]. Accordingly, they possess two organelle specific E3 proteins [42], one is shared between the mitochondrial KADH and the GCS while the other one is apicoplast located and used by PDH only. Its role in *Plasmodium* will be discussed below.

KGDH is a mitochondrial protein complex that catalyses the oxidative decarboxylation of α-ketoglutarate to succinyl-CoA as an integral part of the TCA cycle. Succinyl-CoA is either converted to succinate through the activity of succinyl-CoA synthetase, as part of the TCA cycle, or it is used as a substrate for haem biosynthesis. The NADH generated in the KGDH reaction feeds reducing equivalents and protons into the respiratory chain via complex I (NADH dehydrogenase). *P. falciparum* possesses a KGDH which is possibly located in the mitochondrion.

BCDH is also mitochondrial and is usually involved in the degradation of the branched-chain amino acids valine, leucine and isoleucine. First, the branched-chain amino acid transaminase generates the branched chain α-ketoacids. A gene encoding this protein is missing in the genome of *Plasmodium *but is present in the related apicomplexan parasite* Toxoplasma gondii *[43]. Valine is converted into α-ketoisovaleric acid, leucine into α-ketoisocaproic acid and isoleucine into α-keto-β-methylvaleric acid. These α-ketoacids are subsequently oxidative decarboxylated by BCDH, generating isobutyryl-CoA, isovaleryl-CoA and α-methylbutyryl CoA, respectively. Eventually, the acyl-CoA products can be converted to acetyl-CoA and/or succinyl-CoA and feed into the TCA cycle or other metabolic reactions. Homologues of BCDH-E1α and BCDH-E1β as well as BCDH-E2 were identified in *P. falciparum*, and it was shown that BCDH-E1β is mitochondrial in *Plasmodium*, suggesting that the other components of the enzyme complex are also found in this organelle [43, 44].

The GCS represents another mitochondrial multi-enzyme complex requiring LA as cofactor. It catalyses the oxidative decarboxylation and deamination of glycine, generating CO_2_, NH_3_, NADH and N^5^,N^10^-methylene tetrahydrofolate (CH_2_-THF). The GCS consists of multiple copies of four protein subunits, namely P-protein, H-protein, T-protein and L-protein. The P-protein is a pyridoxal phosphate dependent decarboxylase that catalyses the decarboxylation of glycine and the reductive methylamination of the lipoamide, which is covalently attached to the H-protein. The T-protein requires THF for its activity and catalyses the transfer of methylene to THF and the subsequent release of NH_3_. The H-protein then reacts with the L-protein, the dihydrolipoamide dehydrogenase or E3-subunit described above, to re-oxidise dihydrolipoamide using NAD^+^ as the final electron acceptor. The released CH_2_-THF reacts with another glycine molecule, resulting in the formation of serine, a reaction catalysed by serine hydroxymethyltransferase (SHMT), a protein closely associated with the GCS [22]. The H-protein of *P. falciparum *GCS was located to the mitochondrion [45], confirming that the GCS is likely to be mitochondrial in the parasites. Overall, the reaction mechanism of the GCS is very similar to that of the KADHs, with LA bound to the H-protein functioning as a shuttle transferring reaction intermediates between the active sites of the enzymes in the complex. Structurally, the H-protein is related to the KADH-E2 lipoyl-domains and can be considered as the "lipoyl-domain" of the GCS [22].

## 4 LIPOIC ACID IS A CO-FACTOR OF MITOCHONDRIAL AND APICOPLAST LOCATED MULTI-ENZYME COMPLEXES IN *PLASMODIUM*

As outlined above, *Plasmodium *possesses three KADHs; PDH, KGDH and BCDH as well as the GCS [17, 43, 46, 47]. GCS, KGDH and BCDH are located in the mitochondrion while the sole PDH is located exclusively in the apicoplast [41, 44, 45, 48]. This is an unusual metabolic situation and a recent study provided evidence that *P. falciparum* utilises an unusual branched TCA metabolism with α-ketoglutarate rather than pyruvate as entry point. α-Ketoglutarate is either oxidised or reduced, which allows the formation of acetyl-CoA for metabolic processes such as acetylation reactions, and it was found that malate is an end product of TCA metabolism [49]. This requires the activity of KGDH and accordingly, it was found that the E2-subunit of the enzyme complex is lipoylated [50, 51]. The role of BCDH in *Plasmodium *is not clear, but its E2-subunit is also found to be lipoylated, suggesting that the protein complex or at least the E2-subunit is catalytically active [50, 51]. In *T. gondii* BCDH may be a source for acetyl-CoA in the mitochondrion by catabolising branched-chain amino acids. A gene encoding branched-chain amino acid transaminase (BCAT) was identified in *T. gondii*, but appears to be absent form *Plasmodium* [43]. α-Ketoacids generated from valine, isoleucine or leucine via BCAT are further catabolised through the BCDH in *T. gondi*. A by-product of the acetyl-CoA production through this process is the generation of propionyl-CoA, a metabolite that inhibits cell growth unless detoxified via the methylcitrate cycle. Genes encoding proteins involved in this metabolic activity have been identified in *T. gondii *and other apicomplexan parasites, but they are lacking from the *Plasmodium *genome [43]. The presence of a lipoylated form of BCDH-E2 in *Plasmodium* suggests however, that the BCDH enzyme complex may be active during their intra-erythrocytic life but, as outlined above, it is unlikely to be involved in the catabolism of branched-chain amino acids. It has been suggested that *Plasmodium *BCDH utilises pyruvate as a substrate to generate acetyl-CoA; this metabolic activity may be relevant at times of high demand for acetyl-CoA. This hypothesis is supported by the finding that BCDH from other organisms also has a less pronounced substrate specificity and catabolises pyruvate, albeit with low efficiency [52, 53]. 

The role of the GCS is also not entirely clear in *Plasmodium*, as only genes encoding T-protein, H-protein and L-protein have been identified to date while no homologue of P-protein appears to be present in the parasite [43, 54]. Lipoylated H-protein is readily detectable in *P. falciparum* [50, 51], suggesting a metabolic role of the protein in the mitochondrion [45]. The GCS is associated with SHMT and two forms of this enzyme have been described in* P. falciparum*. Cytosolic SHMT has been recombinantly expressed and was found to be catalytically active, but the proposed mitochondrial protein was inactive [55-57]. Therefore, further investigation is needed to elucidate the role of GCS in one-carbon metabolism in *Plasmodium*. PDH is located in the apicoplast and its major role is to produce acetyl-CoA as substrate for the apicoplast resident FAS II [41, 46, 58, 59].

With this organelle-specific distribution of the KADHs and the GCS, LA must be available in both mitochondrion and apicoplast to guarantee activity of the multi-enzyme complexes [44, 48]. Potentially LA also has an antioxidant role in *Plasmodium* given its prominent redox and antioxidant function described in other organisms (see above). 

LA is a dietary component and mammalian cells take it up through a Na^+^-dependent multivitamin transporter or a proton-linked monocarboxylic acid transporter [60, 61]. It is not clear how *Plasmodium *salvage LA from their host, but it is possible that LA is taken up by the parasitized erythrocyte through the pantothenate tranporter, recently characterised by Saliba and colleagues [62, 63]. Salvaged LA is ligated to KADH and GCS by either the mammalian-like or bacteria-like salvage pathways [64-66]. The pathways operational in *Plasmodium* will be discussed below. LA is also synthesised *de novo *in mammals, bacteria, yeast, plants and parasitic protozoa by the action of two enzymes: octanoyl-acyl carrier protein : protein N-octanoyltransferase (LipB) and lipoate synthase (LipA) [67-69]. The LA biosynthesis pathway is normally mitochondrial, while in plants the pathway is also found in the chloroplast [67]. In *Plasmodium* and *T. gondii*, LA *de novo* biosynthesis is restricted to the apicoplast [70-72] and LA salvage is confined to the mitochondrion [48, 51, 72]. 

## 5 LIPOIC ACID BIOSYNTHESIS IN *PLASMODIUM*

**Fig. (3):**
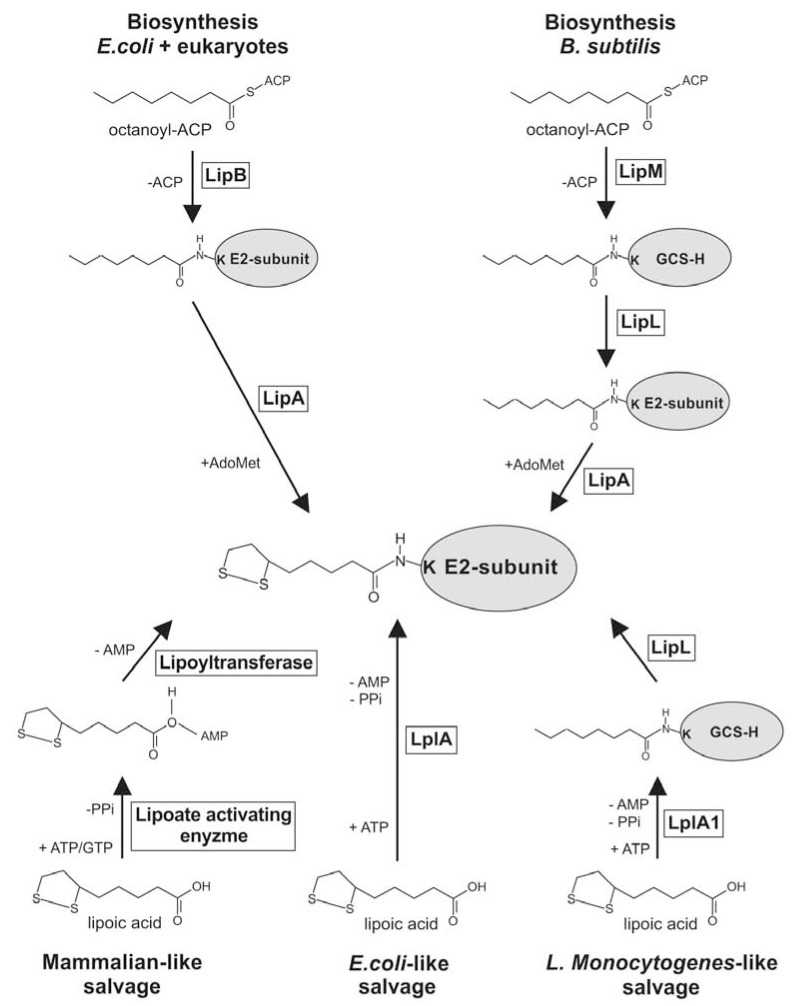
**Lipoic acid salvage and biosynthesis pathways**. α-Lipoic acid is generated and attached to the E2-subunit of KADH or the H-protein of GCS by two mechanisms. In *E. coli* and eukaryotes (including *Plasmodium*), LipB transfers the octanoyl-moiety from Oct-ACP directly to the lipoyl-domain of E2 and two sulphurs are subsequently introduced by LipA. In *B. subtilis* the LipB homologue LipM transfers the ocantoyl-moiety to the H-protein of the bacterial GCS. Octanoylated GCS-H protein is the substrate for the transamidase LipL, which transfers the octanoyl-moiety to the lipoyl-domain of the E2-subunit which is followed by the introduction of the sulphurs via LipA. It is not fully understood if LipA functions before or after the action of LipL. Salvage of α- lipoic acid is achieved through the activity of two proteins (lipoate activating enzyme and lipoyltransferase) in mammals while *E. coli* (and *Plasmodium*) only requires the activity of LplA to catalyse the ligation of free α-lipoic acid to the lipoyl-domain of the E2-subunit of KADH or the H-protein of the GCS. In *L. monocytogenes*, LplA1 catalyses the ligation of α-lipoic acid to the GCS-H-protein before it is transferred by LipL to the E2-subunit of the KADH.

In *Escherichia coli* LA biosynthesis has been extensively studied. It was shown that LA biosynthesis is initiated by LipB, which transfers the octanoyl-moiety from octanoyl-acyl carrier protein (Oct-ACP), an intermediate of FAS II, to the apo-E2-subunit of KADH or to the H-protein of the GCS [66]. Following this reaction two sulphurs are introduced into position C6 and C8 of the octanoyl-moiety by LipA, an S-adenosylmethionine dependent, [Fe-S] cluster containing enzyme that belongs to the SAM-radical family of proteins [73, 74]. LipB is highly specific for its thioester substrate and is unable to transfer salvaged, free LA to the apo-proteins [66, 75]. Recent studies on the *de novo* biosynthesis pathway of LA in *Bacillus subtilis *revealed that these bacteria employ a mechanism in which the LipB homologue, called LipM, transfers the octanoyl-moiety from Oct-ACP to the H-protein of the GCS. Octanoylated H-protein is subsequently used as a substrate for LipL, which catalyses the transamidation of the octanoyl-moiety from H-protein to the apo-E2-subunit of PDH. LipL forms a thioester-linked acyl-enzyme intermediate and probably uses a Cys-Lys dyad for catalysis. The sulphurs are introduced by a LipA homologue, either before or after the LipL reaction [76, 77] (Fig. **3**). A similar reaction cascade appears to operate in yeast, where Lip2 and Lip5 (homologues of LipB and LipA) are sufficient to lipoylate the H-protein of the GCS, while a third protein called Lip3 is required for the lipoylation of PDH and KGDH. It has also been postulated that lipoylated H-protein may be the donor of the lipoyl-moiety that is transferred to PDH and KGDH [78].

Current knowledge suggests that in* Plasmodium *and *Toxoplasma*, LA biosynthesis is performed by the action of LipB and LipA, similar to the situation in *E. coli* (Fig. **3**) [70, 72], and takes place in the apicoplast, where PDH is lipoylated [71]. Accordingly, in *T. gondii* inhibition of FAS II, the source of Oct-ACP, only affects lipoylation of PDH and has no impact on the lipoylation of the mitochondrial KADHs, demonstrating that *Toxoplasma* generates LA in the apicoplast and that this is solely utilised to lipoylate PDH. The drastic reduction of PDH lipoylation also supports the hypothesis that salvaged LA cannot enter the apicoplast and compensate for the absence of LA biosynthesis. On the other hand it was shown that applying 8-bromo-octanoic acid (8-BOA), an inhibitor of LA salvage, resulted in the loss of mitochondrial KADH and H-protein lipoylation while PDH lipoylation was maintained [71]. Conditional down-regulation of FAS II in *T. gondii *resulted in the loss of PDH lipoylation and was lethal for *T. gondii *[79], suggesting that LA biosynthesis is vital for these apicomplexans. Therefore it was presumed that LA biosynthesis should also be crucial for the survival of *Plasmodium*, but a disruption of the *lipB* gene in the erythrocytic stage of *P. falciparum* had no effect on parasite viability. By contrast the progression through their intra-erythrocytic cell cycle was moderately accelerated [50]. The main phenotype of the *lipB* null mutant was a drastic reduction of the total (reduced and oxidized) LA content by ~95%, resulting in a reduced lipoylation of the PDH E2-subunit. The remaining lipoylation of PDH was thought to be the result of the activity of one of the lipoic acid protein ligase-like proteins present in the parasites (LplA 2), that was shown to be dually targeted to the apicoplast and the mitochondrion [50]. In agreement with these results neither FAS II nor PDH have essential functions during the intra-erythrocytic development of *Plasmodium*, but they were found to be vital for liver stage development [59, 80, 81]. Therefore it can be postulated that this is also the case for LA biosynthesis. 

Overall it can be concluded that in *Plasmodium* the LA biosynthesis pathway is not a “classical” drug target whose inhibition would lead to the prevention of the intra-erythrocytic development and thus cure patients from malaria. However, the phenotypes of *P. yoelli* *pdh* and *fabB/F *or *fabZ* null mutants [81], which show developmental arrest of late liver stages, opens new avenues for vaccine development [82, 83] or for exo-erythrocytic chemotherapies. In support of this hypothesis it was found that genetically attenuated *Plasmodium *lacking the FAS II activity induced a more pronounced protective immunity in animal models than irradiated sporozoites. In addition, it was found that they lead to a high level of cross-stage and cross-species protection, which is very exciting and may help in the quest for new antimalarial therapies [84]. 

FAS II is essential for *T. gondii*, a parasite that infects almost all nucleated cells, which suggests that its requirement for fatty acids are not met solely by uptake from its host cell. A number of studies provide evidence that *T. gondii* is able to commandeer metabolites including lipids and its precursors from its host cell [85, 86]. Further, the *T. gondii* genome contains one gene encoding FAS I and possibly two genes encoding polyketide synthase [87], suggesting that these parasites need a more elaborate set of fatty acids and lipids than for instance the malaria parasite during its erythrocytic and sexual development. FAS I also occurs in *Cryptosporidium* and *Eimeria* [87, 88] and might be linked to the formation of oocysts that are shed into the environment, but is absent from *Plasmodium *and *Theileria* [87]. It is possible that *Plasmodium* during liver stage development finds itself in a similar situation as *T. gondii* and the supplies of fatty acids from its host cell are insufficient to support the extensive multiplication it undergoes in the liver. This is surprising given that the liver is the predominant site of fatty acid metabolism in mammals and presumably should provide a sufficiently high supply of the building blocks for parasite lipids. It is known that lipoproteins are required for a successful liver stage infection and UIS3 has been shown to be involved in lipid or fatty acid uptake across the parasitophorous vacuolar membrane (PVM) [89]. So why does *Plasmodium *require an active FAS II when the host cell is actually providing lipids and fatty acids to the developing schizont? 

The formation of thousands of individual merozoites during the development in the liver requires a vast biosynthesis of membranes in a relatively short period of time. It was shown that the membrane of the developing parasites in the liver invaginates profoundly during the cytomere stage, supporting the need for substantial amounts of lipids [90]. This suggests that the requirements for lipids during this parasite stage is more excessive than during the other developmental stages and potentially explains their dependence on FAS II, PDH activity and presumably LA biosynthesis. Another possibility is that the parasites rely on a specific fatty acid that cannot be supplied in sufficiently high concentrations in the liver and therefore needs to be synthesised *de novo* by FAS II. Such a fatty acid may be required for the formation of their GPI-anchors that are absolutely necessary to attach one of the major surface proteins MSP-1 to the merozoite surface. In fact, MSP-1 expression was not detectable in the *P. yoelli* *pdh* and *fabB/F *null mutants, suggesting a role for FAS II [59, 81]. This is reminiscent of findings that *Trypanosoma brucei* blood stream form parasites rely on large amounts of myristic acid to guarantee the correct lipidation of their GPI anchors [91]. The amount of myristate provided by uptake is insufficient and these parasites make use of a specific elongase-dependent FAS system to generate the required amounts of this fatty acid.

## 6 LIPOC ACID SALVAGE IN *PLASMODIUM*

*P. falciparum*, *T. gondii* and other apicomplexan parasites are not only able to synthesize LA *de novo* but they also scavenge free LA, which is solely used to lipoylate mitochondrial KADH and GCS [51, 71, 72, 92].

How *Plasmodium *acquires LA from its host is unclear, but as mentioned above, LA may be taken up via a pantothenate transporter [62] similar to the situation in mammals [60]. Human serum contains about 33 to 145 ng/ml lipoate, which is non-covalently bound to albumin and available for the intra-erythrocytic stages of *Plasmodium* [93, 94]. The situation for *T. gondii* is quite different because these parasites live in nucleated host cells, which are likely to utilise the scavenged LA for their own metabolic activities [71]. It is possible that *T. gondii* is able to take up lipoylated peptides, resulting from the degradation of host mitochondrial E2-subunits of KADH, as a source of LA, as long as the parasite possesses a lipoamidase activity to release LA from these peptides. Lipoamidase activity has been reported from bacterial and mammalian sources with a specificity for mainly lipoylated peptides or proteins, so that it is conceivable that either *T. gondii* themselves or their host cell provide LA for lipoylation reactions [95-100]. This hypothesis is particularly appealing for *T. gondii* because the PVM forms a tight association with the host cell mitochondria and endoplasmic reticulum and metabolites are potentially provided from the host organelles to the parasite [71, 101]. In addition, *T. gondii *sequesters host endolysosomes in their parasitophorous vacuole via host microtubules [102], which also could be a source of free LA. This is certainly not the case in *P. falciparum*-infected erythrocytes, albeit a similar situation might occur during liver stage development [103]. It was shown that the PVM during *Plasmodium *liver stage development becomes porous and allows passive transfer of molecules up to 855 kDa in size [104], which would allow LA-containing host peptides to be scavenged by the parasites. A similar process was reported for the acquisition of LA by the intracellular bacterium *Listeria monocytogenes *[105]. 

Lipoic acid protein ligases or LplAs catalyze a two-step reaction: first, LA is activated by ATP, generating the LA-AMP intermediate that remains bound to the active site of the enzyme while pyrophosphate is released. In the second step the transfer of LA to the lipoyl-domain of the apo-target protein occurs and AMP is released [65, 66, 106]. This two-step reaction, catalyzed by a single LplA protein in *E. coli *and *Plasmodium*, requires the activity of two enzymes in mammalian cells: a lipoate activating enzyme and a lipoyltransferase (Fig. **3**) [64, 65, 106]. Recently it was found that *Thermoplasma acidophilum* also needs two enzymes to lipoylate its KADH. In these bacteria two Lpl proteins, LplA and LplB, form a heterodimer which displays LA ligase activity [107]. 

**Fig. (4):**
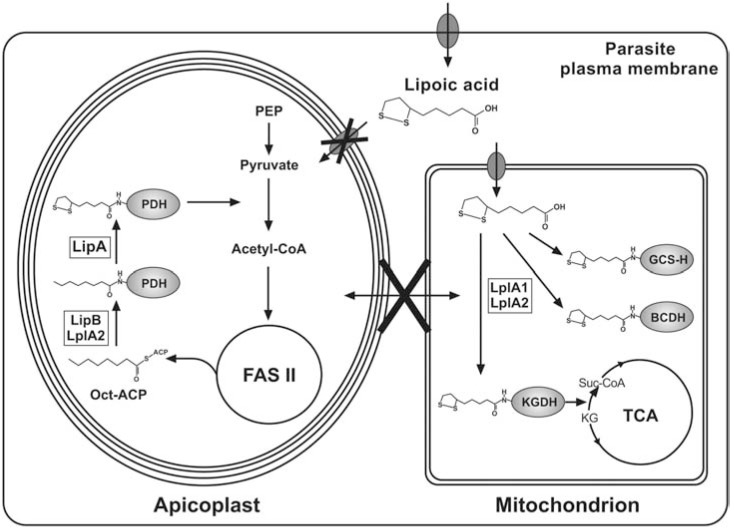
**Lipoic acid metabolism in *Plasmodium***. Biosynthesis and ligation of LA to PDH takes place in the apicoplast. Oct-ACP is an intermediate of FAS II and is attached to the E2-subunit of PDH by LipB or LplA2, followed by the insertion of two sulphurs by LipA, generating the PDH-bound lipoyl-moiety. PDH converts pyruvate into acetyl-CoA, which feeds into FAS II. Free LA cannot enter the apicoplast and is exclusively used for the lipoylation of mitochondrial complexes. LA is activated and ligated to the H-protein of the GCS (GCS-H) and the E2-subunit of BCDH and KGDH by LplA1 or LplA2. KGDH converts α-ketoglutarate (KG) into succinyl-CoA as part of the branched TCA metabolism. LA is possibly taken up by the parasite via a pantothenate transporter, located in the parasite plasma membrane.

*Plasmodium* and *Toxoplasma *possess two genes encoding LplA-like proteins, LplA1 and LplA2. *Plasmodium* LplA1 has modest sequence similarly to *E. coli *LplA, while LplA2 seems to be divergent from both the *E. coli* LplA and the *Plasmodium *LplA1 [47, 50, 51]. LplA1 and LplA2 of *P. falciparum* complemented the growth defect of an *E. coli *mutant lacking *lplA *and* lipB* demonstrating that both have LplA activity [50, 51, 72]. *Plasmodium* LplA1, recombinantly expressed in *E. coli*, was shown to be enzymatically active and preferably lipoylates H-protein, while the lipoyl-domains of the apicoplast PDH are poor substrates, which is in agreement with LplA1’s location in the parasite’s mitochondrion [51, 72]. LplA2 appears to be present in both mitochondrion and apicoplast and it can replace the function of apicoplast LipB, as discussed above, but not the function of the mitochondrial LplA1 [50, 92] (Fig. **4**). *L*. *monocytogenes *also possesses two LplA proteins, similar to *Plasmodium,* but the bacteria lack the ability to synthesise LA *de novo* and are LA auxotrophs [108]. Interestingly, only one of the ligases (LplA1) is important for intracellular growth of the bacteria *in vivo *and it has been shown that its role is to scavenge LA that is provided by LA-modified host cell peptides [105]. The role of *L. monocytogenes *LplA2 is uncertain, but it was recently shown that the recombinant protein complements an *E. coli *line deficient in LA salvage and biosynthesis, demonstrating that it is an authentic LplA ligase [109]. Similar to *B. subtilis, L. monocytogenes *encodes a LipL homologue that acts as an amidotransferase that operates in conjunction with LplA1 (and not LipB as in *B. subtilis)*. *L. monocytogenes *LplA1 preferentially lipoylates H-protein of the bacterial GCS. This is then used by LipL to transfer the lipoyl-moiety from H-protein to the E2-subunits of KADH [109]. However, the complexity of the lipoylation pathways recently discovered in bacteria may not be the same in eukaryotes and it remains to be seen whether LplA2 in *Plasmodium* has an accessory function rather than being a *bona fide* lipoic acid protein ligase as the complementation assays suggest (Fig. **4**). 

The presence of two LplA proteins in *Plasmodium *suggested potential redundancy of the two proteins but a reverse-genetics approach to disrupt either of the two genes in *P. falciparum* was unsuccessful. The gene loci appeared refractory to recombination as it was impossible to target them with knockout as well as knock-in constructs [92]. This is clearly an unsatisfactory situation and allows little conclusion as to the validity of these proteins for future drug development. Therefore it was attempted to generate a *lplA1* gene deletion in *P. berghei*, where the gene locus was targeted by knock-in and knock-out constructs. Despite replacing the* lplA1* gene with a selectable marker, it was impossible to isolate a stable population of the null mutant parasites [92], suggesting that the protein is important for growth and survival of the erythrocytic stages of the malaria parasites. This hypothesis is supported by results from inhibition studies using the LA analogue 8-BOA that negatively affected intra-erythrocytic growth of *P. falciparum* [51], indicating that LplA proteins play an essential role for the development of these *Plasmodium* stages. Similarly it was demonstrated that LA salvage is essential for *P. berghei* during liver stage development [103]. Elimination of lipoylation of mitochondrial KADH, using 8-BOA, had a negative impact on parasite development, while lipoylation of apicoplast PDH remained unchanged.

In addition to the importance for the function of the parasite’s KADH and GCS, it has been suggested that free or protein-bound LA has antioxidant and redox regulating properties. In *Mycobacteria*, protein-bound LA interacts with a thioredoxin-like protein and, protects the bacteria from unwanted and harmful reactive oxygen species [35]. Similarly, DHLA bound to mammalian KGDH reduces thioredoxin [110] and in *E. coli* protein-bound DHLA serves as a source of electrons for the reduction of glutaredoxins [36]. Mounting evidence suggests that free and protein bound LA are critically involved in redox regulation [28, 111, 112]. However, it remains for elucidation whether this is also the case in *Plasmodium*. The likelihood of protein-bound LA being involved in redox regulation in the apicoplast during intra-erythrocytic development of the parasites is negligible. A recent study demonstrated unambiguously that LA biosynthesis is not important for apicoplast maintenance and that LA generated by the LA biosynthetic pathway is not essential for parasite survival. It appears that the only vital pathway present in the organelle is the non-mevalonate isoprenoid biosynthesis [19]. 

## 7 CONCLUSIONS

LA is a crucial component of metabolism of the malaria parasite and may also be involved in maintenance of its organellar redox homeostasis. Its salvage and biosynthesis offer potential for the development of future antimalarial drugs and vaccines 

The investigations of the unusual, organelle-specific distribution of LA *do novo *biosynthesis and salvage pathways in *Plasmodium *have revealed that both the apicoplast LA biosynthesis and mitochondrial salvage pathways are essential for the development of the parasites in its human host. The inability to generate viable *lplA*1 null mutants of *P. falciparum *and* P. berghei* together with the findings that 8-BOA inhibits *P. falciparum* progression through the intra-erythrocytic cycle as well as* P. berghei *liver stage development, strongly support that the pathway offers potential for drug development against malaria*. *The LA salvage reaction operating in *Plasmodium *differs considerably from that present in the human host, indicating that specific inhibition of the parasite LA salvage pathway may be feasible. Clearly a full biochemical characterisation and structural analysis of *Plasmodium *LplA proteins would facilitate parasite-specific drug design in the future.

LA *de novo* biosynthesis is not of critical importance during intra-erythrocytic growth of *P. falciparum*, but circumstantial evidence (the importance of FASII and PDH for the liver stage) suggests that it may be vital for the development of the parasite in the liver. The fact that the human host also possesses the LA biosynthetic pathway similar to that of *Plasmodium *reduces the feasibility that the parasite pathway can be targeted by inhibitors specifically. Mammalian cells salvage LA efficiently but they rely on LA biosynthesis during embryonic development [69]. Since one major aim of antimalarial drug development strategies is to prevent and treat disease in young children and pregnant women, who are most affected by malaria, LA biosynthesis of *Plasmodium *may not be the ideal drug target to pursue for general treatment of the disease. On the other hand, LA salvage offers great potential as target for both blood and liver stage *Plasmodium* and may be worthwhile following up in future preventative drug and possibly vaccine development programmes.

The genes/proteins mentioned in text have the following identification numbers and have been identified with PlasmoDB (http://plasmodb.org/plasmo/) for the *Plasmodium* genes and with ToxoDB (http://toxodb.org/toxo/) for the *Toxoplasma* genes. For the other organisms NCBI-DB was used.

###  P. falciparum:

LipA: MAL13P1.220, LipB: Mal8P1.37, LplA1: PF13_0083, LplA2: PFI1160w, ACP: PFB0385w, BCDH-E2: PFC0170c, BCDH-E1α: PF13_0070, BCDH-E1β: PFE0225W, KGDH-E2: PF13_0121, PDH-E2: PF10_0407, H-protein: PF11_0339, T-protein: PF13_0345, L-protein: PFL1550W, cytosolic SHMT: PFL1720w, mitochondrial SHMT: PF14_0534, FabI: PFF0730c, pantothenate transporter: PF11_0059.

### P. berghei:

LplA1: PB000283.02.0, LplA2: PB001158.00.0.

### P. yoelli:

PDH-E1α:PY00819, PDH-E3: PY00573, FabB/F: PY04452, FabZ: PY01586, FabI: PY03846.

### T. gondii:

LipA: 42.m00009, LipB: 583.m05698, LplA1: 59.m00073, LplA2 83.m01296, BCAT: 113.m01283, FabI: 50.m00011, FAS I: 83.m00010, putative polyketide synthase: 20.m03875 and 46.m01725.

### E. coli:

LipA: AAB40828, LipB: NP_415163, LplA: NP_418803.

### L. monocytogenes:

LplA1: CAC99009, LplA2: CAC98842, LipL: CAD00644, H-protein: Q8Y4L2.

### T. acidophilum:

LplA: CAC11654, LplB: NP_393990.

### B. subtilis:

LipM: BAA12550, LipA: 032129, LipL: P39648, H-protein: 032174.

### S. cerevisiae:

Lip2: NP_013340, Lip5: CAA99409, Lip3: P47051, H-protein: AAC04987.

### Bos taurus:

lipoate-activating enzyme: BAB40420, lipoyltransferase: BAA24354.

## ABBREVIATIONS

ACP: = Acyl carrier protein
ACT: = Artemisinin combination therapy
BCDH: = Branched chain α-ketoacid dehydrognease
CH_2_-THF: = N^5^,N^10^-methylene tetrahydrofolate
CoA: = Coenzyme A
DHLA: = Dihydrolipoic acid
FAS II: = Type II fatty acid biosynthesis
GCS: = Glycine cleavage system
KADH: = α-ketoacid dehydrogenase complex
KGDH: = α-ketoglutarate dehydrogenase complex
LA: = α-lipoic acid
LipA: = Lipoic acid synthase
LipB: = Octanoyl-acyl carrier protein (ACP): protein N-octanoyltransferase
LplA: = Lipoic acid protein ligase
Oct-ACP: = Octanoyl-acyl carrier protein
PDH: = Pyruvate dehydrogenase complex
PVM: = Parasitophorous vacuole membrane
SHMT: = Serin hydroxymethyltransferase
TCA: = Tricarboxylic acid
8-BOA: = 8-bromoocatnoic acid
